# Viral Mediated Redirection of NEMO/IKKγ to Autophagosomes Curtails the Inflammatory Cascade

**DOI:** 10.1371/journal.ppat.1002517

**Published:** 2012-02-02

**Authors:** Patricia M. Fliss, Tali Pechenick Jowers, Melanie M. Brinkmann, Barbara Holstermann, Claudia Mack, Paul Dickinson, Heinrich Hohenberg, Peter Ghazal, Wolfram Brune

**Affiliations:** 1 Heinrich Pette Institute, Leibniz Institute for Experimental Virology, Hamburg, Germany; 2 Division of Viral Infections, Robert Koch Institute, Berlin, Germany; 3 Division of Pathway Medicine, Centre for Infectious Diseases, University of Edinburgh Medical School, Edinburgh, United Kingdom; 4 Helmholtz Center for Infection Research, Braunschweig, Germany; University of North Carolina at Chapel Hill, United States of America

## Abstract

The early host response to viral infections involves transient activation of pattern recognition receptors leading to an induction of inflammatory cytokines such as interleukin-1β (IL-1β) and tumor necrosis factor α (TNFα). Subsequent activation of cytokine receptors in an autocrine and paracrine manner results in an inflammatory cascade. The precise mechanisms by which viruses avert an inflammatory cascade are incompletely understood. Nuclear factor (NF)-κB is a central regulator of the inflammatory signaling cascade that is controlled by inhibitor of NF-κB (IκB) proteins and the IκB kinase (IKK) complex. In this study we show that murine cytomegalovirus inhibits the inflammatory cascade by blocking Toll-like receptor (TLR) and IL-1 receptor-dependent NF-κB activation. Inhibition occurs through an interaction of the viral M45 protein with the NF-κB essential modulator (NEMO), the regulatory subunit of the IKK complex. M45 induces proteasome-independent degradation of NEMO by targeting NEMO to autophagosomes for subsequent degradation in lysosomes. We propose that the selective and irreversible degradation of a central regulatory protein by autophagy represents a new viral strategy to dampen the inflammatory response.

## Author Summary

Upon viral infection cells immediately induce an innate immune response which involves the production of inflammatory cytokines. These cytokines activate specific receptors on infected and surrounding cells leading to local signal amplification as well as signal broadcasting beyond the original site of infection. Inflammatory cytokine production depends on transcription factor NF-κB, whose activity is controlled by a kinase complex that includes the NF-κB essential modulator (NEMO). In order to replicate and spread in their hosts, viruses have evolved numerous strategies to counteract innate immune defenses. In this study we identify a highly effective viral strategy to blunt the host inflammatory response: The murine cytomegalovirus M45 protein binds to NEMO and redirects it to autophagosomes, vesicular structures that deliver cytoplasmic constituents to lysosomes for degradation and recycling. By this means, the virus installs a sustained block to all classical NF-κB activation pathways, which include signaling cascades originating from pattern recognition receptors and inflammatory cytokine receptors. Redirection of an essential component of the host cell defense machinery to the autophagic degradation pathway is a previously unrecognized viral immune evasion strategy whose principle is likely shared by other pathogens.

## Introduction

Transcription factor NF-κB activates the expression of numerous target genes, most of which are involved in regulating innate and adaptive immune responses [1], [2]. It is activated in response to a variety of stimuli, which include pathogen-associated molecular patterns (PAMPs) and proinflammatory cytokines, such as TNFα and IL-1β. While TNFα and IL-1β activate their specific receptors at the cell surface, PAMPs are recognized by so-called pattern-recognition receptors (PRRs) located at the cell surface, within endosomal membranes, or the cytosol [3]. The best characterized PRRs are the TLRs, a family of transmembrane proteins that recognize PAMPs at the cell surface or within endosomes [4]. They detect a broad range of PAMPs originating from viruses, bacteria or fungi. For instance, TLR2 and 4 are typically activated by bacterial peptidoglycans and lipopolysacharide (LPS), respectively. However, they can also be activated by certain viral glycoproteins [5]. Other TLRs, such as TLR3, 7, and 9, recognize double- or single-stranded RNA or unmethylated DNA of viral or bacterial origin [6].

The NF-κB activation pathways emanating from IL-1 receptor (IL-1R), TNF receptor 1 (TNFR1), and PRRs such as the TLRs are similar and overlapping (Figure S1). In all these pathways, NF-κB activity is controlled by inhibitory IκB proteins, of which IκBα is the best-characterized, and by the IκB kinase (IKK) complex. The IKK complex consists of two catalytic subunits, IKKα and β [7], and the essential regulatory subunit, IKKγ, which is more commonly referred to as NEMO (NF-κB essential modulator) [8]. NEMO acts as a scaffold protein for the IKK complex and mediates interactions with upstream signaling molecules such as RIP1 and IRAK1 [9], [10]. Upon activation, the IKK complex phosphorylates IκBα, resulting in a rapid ubiquitylation and proteasomal degradation of IκBα. By this means, NF-κB is released from its inhibitor, translocates to the nucleus, and activates transcription of proinflammatory cytokines, chemokines, and antiapoptotic and antimicrobial proteins [11], [12].

During viral infection the first wave of proinflammatory cytokine production is induced by PRRs upon virus recognition [5], [13]. This immediate and transient response is sustained and further potentiated by cytokines, such as TNFα and IL-1β, which activate their cognate receptors in an autocrine and paracrine manner (Figure S1). This allows for local signal amplification as well as systemic signal broadcasting beyond the original site of infection [14], [15]. The ensuing inflammatory cascade further promotes vascular endothelial permeability and infiltration of leukocytes to the site of infection, and is key to both pathogen elimination and tissue healing [16].

Cytomegaloviruses are large DNA viruses of the herpesvirus family that are highly prevalent and cause lifelong infections in their respective host populations [17]. They induce a broad spectrum of innate and adaptive immune responses including the production of cytokines, induction of programmed cell death, and priming of T lymphocytes, but have also evolved various strategies to modulate these antiviral host responses. Co-evolution of these viruses with their hosts resulted in a dynamic equilibrium between the host immune response and viral immune evasion strategies [18]. However, when this equilibrium gets out of balance, as it is the case in immunosuppressed transplant recipients and immunologically immature fetuses, serious disease may ensue [17].

Human and murine cytomegalovirus (HCMV and MCMV) both manipulate the early inflammatory host response by interfering with the NF-κB pathway: they first induce and later inhibit NF-κB activation. Initially, NF-κB is activated following viral attachment and entry into host cells [19], [20] leading to the production of cytokines. This activation is triggered, at least in part, by viral envelope glycoproteins interacting with TLR2 [21], and viral DNA stimulating TLR9 [22], [23], [24]. TLR3 and TLR7 are also involved in sensing CMV infection [22], [24], although it is not clear whether they are activated during initial infection or later during the replication cycle. Besides TLRs, CMV also stimulates cytosolic DNA sensors. Engagement of ZBP1/DAI activates NF-κB and cytokine production [25], [26], while AIM2 activates caspase-1, which converts the IL-1β precursor into a functional cytokine [27] (Figure S1).

Initial NF-κB activation is thought to produce favorable conditions for viral replication, particularly since the viral major immediate-early promoter contains numerous NF-κB response elements [28]. However, it remains controversial whether or not these NF-κB response elements are necessary to kick-start viral transcription and replication [29], [30], [31]. Later during infection, cytomegaloviruses inhibit NF-κB activation, thereby dampening the inflammatory host response [20], [32]. Specifically, it was shown that IL-1β-mediated NF-κB activation is inhibited in HCMV-infected cells, possibly by interfering with signal transduction upstream of the IKK complex [33]. Whether MCMV also blocks IL-1β signaling has not yet been investigated. HCMV and MCMV also inhibit NF-κB activation in response to TNFα, and this inhibition has been correlated to a down-regulation of TNFR1 from the cell surface [33], [34], [35].

More recent work has shown that the MCMV M45 protein blocks TNFR1- and TLR3-dependent NF-κB activation by interacting with receptor-interacting protein 1 (RIP1), a crucial mediator protein within these signaling pathways [36]. M45 also inhibits TNFR1-dependent activation of p38 mitogen-activated protein kinase (MAPK) and programmed necrosis [36]. For the latter, a RIP homotypic interaction motif (RHIM) within M45 was shown to be essential [37]. The M45 RHIM is required to inhibit the interaction of RIP1 with RIP3, which is necessary for TNFα-induced necrosis, and is also required for preventing RIP3-mediated necrosis in response to other stimuli [38].

Here we show that the cytomegalovirus M45 protein blocks TLR- and IL-1R-dependent NF-κB activation and cytokine production by targeting the IKK complex, the converging point of all classical NF-κB activation pathways. M45 binds to NEMO and relocalizes it to autophagosomes for subsequent lysosomal degradation. Hence, this virus has adopted an elegant, previously undescribed strategy to blunt the host cytokine response by selectively disposing of an essential regulatory hub protein of the inflammatory cascade.

## Results

### M45 blocks TLR- and IL-1R-dependent NF-κB activation

**Figure 1:**
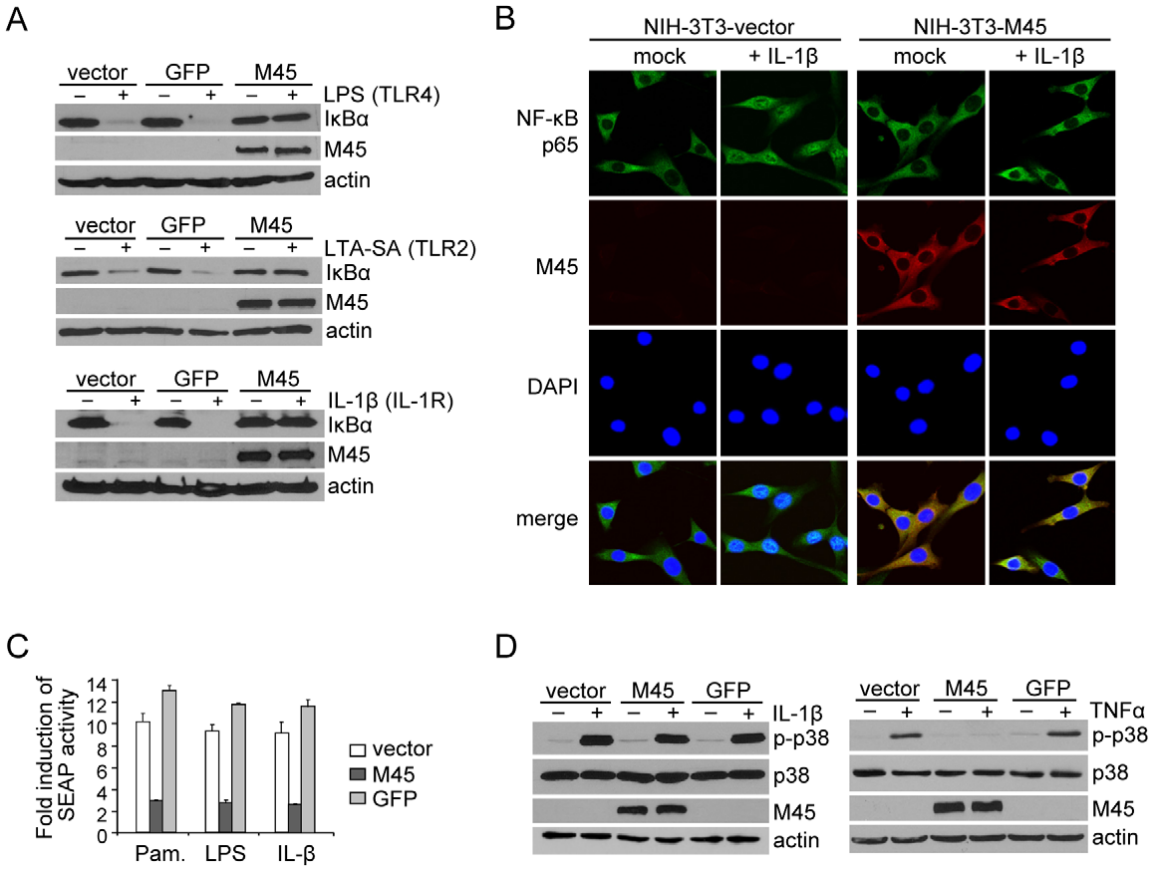
M45 inhibits TLR- and IL-1R-mediated NF-κB but not p38 activation. (A) NIH-3T3 cells were transduced with retroviral vectors expressing M45 or GFP. Two days after transduction cells were stimulated with the TLR4 agonist LPS (10 µg/ml), the TLR2 agonist LTA-SA (10 µg/ml), or IL-1β (20 ng/ml). IκBα levels were determined by immunoblotting. (B) NIH-3T3 cells were transduced with an M45-expressing or an empty retroviral vector. 30 min after stimulation with IL-1β (10 ng/ml) cells were fixed, and subcellular localization of the NF-κB p65 subunit was analyzed by immunofluorescence. (C) NIH-3T3 cells expressing an NF-κB-dependent secreted alkaline phosphatase (SEAP) reporter were transduced with retroviral vectors expressing M45 or GFP and stimulated 78 h later with the TLR2 agonist Pam_3_CSK_4_ (Pam.,1 µg/ml), the TLR4 agonist LPS (0.1 µg/ml), or IL-1β (5 ng/ml). SEAP activity in the supernatant was quantified 15 h after stimulation and is shown as fold induction of SEAP activity of stimulated cells compared to non-stimulated cells (mean ± SD) (D) NIH-3T3 cells transduced with retroviral vectors expressing M45 or GFP were stimulated with IL-1β (20 ng/ml, 15 min) or TNFα (10 ng/ml, 5 min). Phosphorylated and total p38 in cell lysates was detected by immunoblotting.

Previous work has demonstrated that the MCMV M45 protein inhibits NF-κB activation upon TNFR1 or TLR3 stimulation [36]. This inhibitory effect was attributed to the ability of the M45 protein to interact with RIP1 and block RIP1-dependent signaling pathways. Like TLR3, TLR4 can activate NF-κB using the adaptor proteins TRIF and RIP1 (Figure S1). However, TLR4 also utilizes another pathway to activate NF-κB involving the adaptor proteins MyD88, IRAK1, and TRAF6 (Figure S1). Unexpectedly, we found that M45 completely inhibited TLR4-induced degradation of the NF-κB inhibitor IκBα, which was measured as an indicator of NF-κB activation (Figure 1A). Moreover, degradation of IκBα was inhibited after stimulation of TLR2 and IL-1R, which signal exclusively through the MyD88-dependent pathway (Figure 1A). The inhibitory effect correlated with a block of nuclear translocation of the NF-κB p65 (RelA) subunit as shown for stimulation with IL-1β (Figure 1B). M45 also blocked NF-κB activation in an NF-κB-dependent reporter assay upon TLR2, TLR4, and IL-1R stimulation (Figure 1C). However, we could not detect an inhibition of IL-1R-dependent p38 MAPK phosphorylation by M45 (Figure 1D). By contrast, p38 phosphorylation upon TNFR1 stimulation was inhibited (Figure 1D), consistent with previously published data [36].

**Figure 2:**
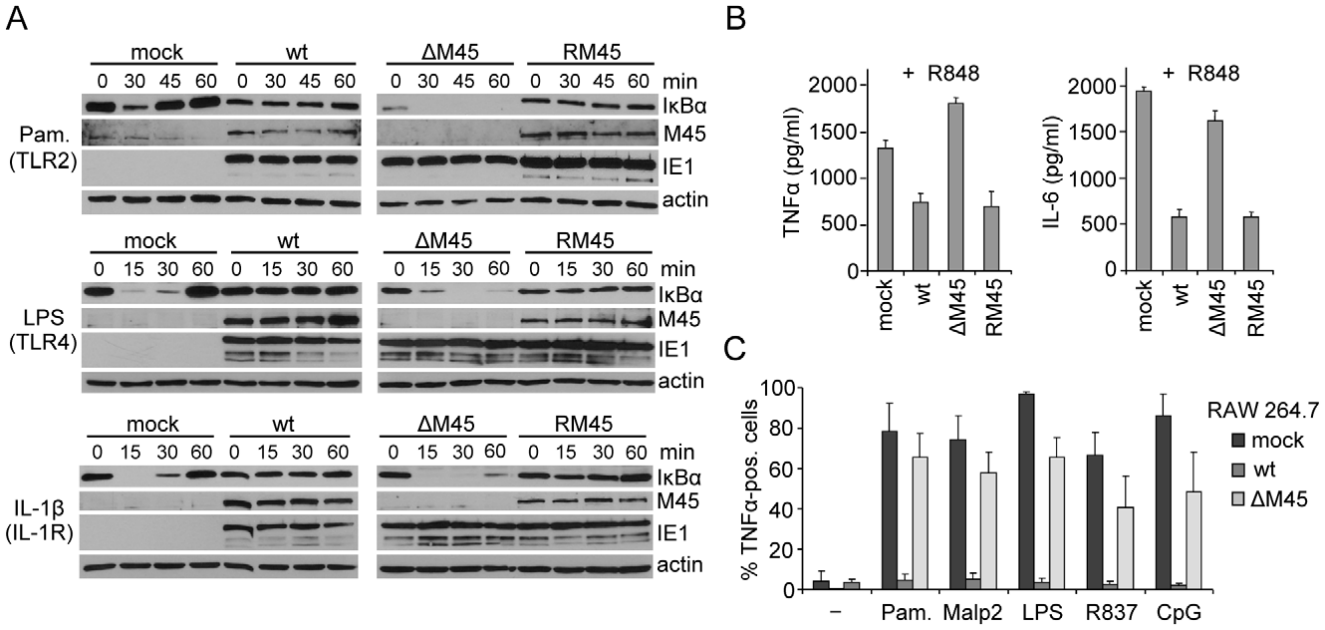
M45 inhibits IκBα degradation, TNFα and IL-6 production during MCMV infection. (A) NIH-3T3 cells were infected with wt MCMV-GFP, an M45 deletion mutant (ΔM45) or a revertant virus (RM45) at an MOI of 10 and treated 5 h postinfection with the TLR2 agonist Pam_3_CSK_4_ (Pam., 0.1 µg/ml), the TLR4 agonist LPS (10 µg/ml), or IL-1β (20 ng/ml) for the indicated times. Levels of the indicated proteins were analyzed by immunoblotting. (B) BMDMs were infected with wt MCMV-GFP, ΔM45, or RM45 at an MOI of 3 and stimulated 8 h postinfection for 16 h with the TLR7 agonist R848 (0.1 µM). TNFα and IL-6 levels in the supernatant were determined by ELISA (mean ± SD). (C) RAW264.7 macrophages were infected with wt MCMV-GFP or ΔM45 at an MOI of 0.1, stimulated 24 h postinfection for 4 hours with TLR agonists Pam_3_CSK_4_ (Pam.) or Malp-2 (TLR2), LPS (TLR4), R837 (TLR7), or CpG (TLR9), in the presence of brefeldin A. Cells were fixed, permeabilized, and stained with a TNFα-specific antibody. The percentages of TNFα-positive cells within infected (GFP-positive) cell populations were determined by FACS analysis (mean ± SD).

As the results shown in Figure 1 were obtained using NIH-3T3 fibroblasts that expressed M45 after transduction with a retroviral expression vector, we next sought to assess whether M45 has the same inhibitory effect in the context of the viral genome during MCMV infection. For these experiments, NIH-3T3 fibroblasts were infected with a GFP-expressing wt MCMV, an M45 deletion mutant (ΔM45), or a revertant virus (RM45). Five hours postinfection (hpi), cells were stimulated with TLR2, TLR4, or IL-1R agonists, and IκBα degradation was determined by immunoblot analysis. As shown in Figure 2A, wt MCMV and the revertant virus inhibited IκBα degradation, but the ΔM45 virus did not.

Macrophages are key immune cells for initial pathogen recognition and production of proinflammatory cytokines. They express a wide range of TLRs not expressed by other cells, such as fibroblasts, including TLR7 and TLR9. Therefore we investigated TLR responses in macrophages. Upon infection of primary bone marrow-derived macrophages (BMDMs), MCMV inhibited nuclear translocation of NF-κB p65 (RelA) after stimulation of TLR7 or TLR9 (Figure S2). Importantly, production of the NF-κB-dependent cytokines TNFα and IL-6 upon TLR7 stimulation was also inhibited in MCMV-infected BMDMs, dependent on the presence of M45 (Figure 2B). Similarly, intracellular accumulation of TNFα upon stimulation with different TLR agonists was inhibited in wt MCMV-infected RAW267.4 macrophages, but not in those infected with the ΔM45 mutant (Figure 2C).

### The M45 protein interacts with NEMO

**Figure 3:**
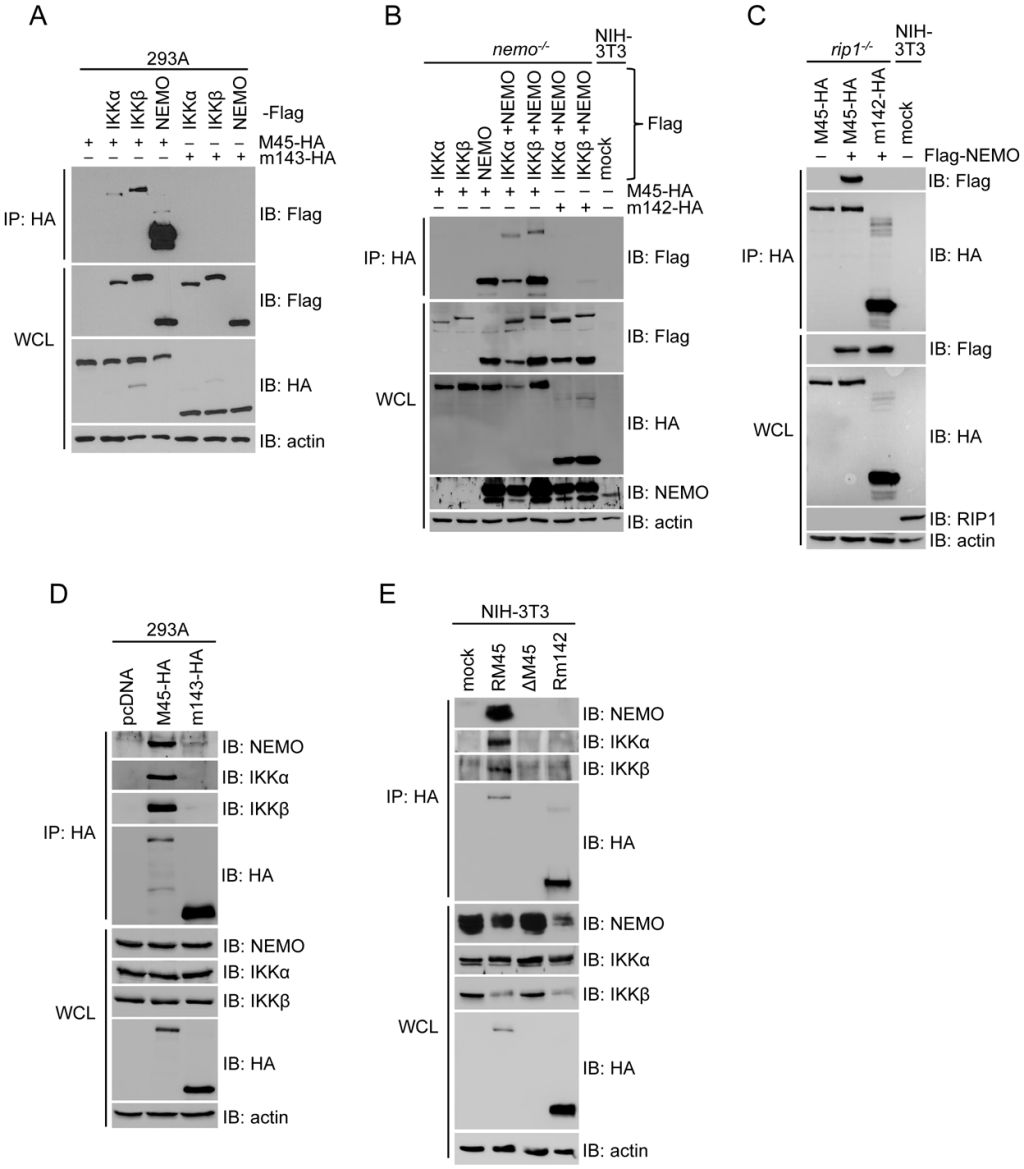
M45 interacts with the IKK complex. (A) 293A cells were transfected with plasmids expressing HA-tagged M45 or an unrelated MCMV control protein (m143) and Flag-tagged IKK subunits. Lysates were subjected to immunoprecipitation (IP) using an anti-HA antibody. Immunoprecipitates were analyzed by immunoblotting (IB) using anti-Flag antibody. Whole cell lysates (WCL) were immunoblotted using Flag-, HA-, and actin-specific antibodies, respectively. (B) *Nemo* ^−/−^ MEFs were transfected with Flag-tagged IKK subunits and HA-tagged M45 or an unrelated MCMV control protein (m142), respectively. IP and IB were performed using anti-HA and anti-Flag antibodies, respectively. WCL were immunoblotted with anti-NEMO antibody and as described for panel A. (C) *rip1* ^−/−^ MEFs were transfected with HA-tagged M45 or an unrelated MCMV control protein (m142) and Flag-tagged NEMO. IP was done with an anti-HA antibody. Immunoprecipitates and WCL were analyzed by immunoblotting using the indicated antibodies. (D) 293A cells were transfected with plasmids expressing HA-tagged M45 or m143 (as a control). Lysates were subjected to anti-HA immunoprecipitation. Immunoblotting of precipitates and the WCL was done with antibodies as indicated. (E) NIH-3T3 cells were infected for 4 hours with RM45 (MCMV expressing HA-tagged M45), ΔM45, or Rm142 (MCMV expressing HA-tagged m142) at an MOI of 5. Cell lysates were subjected to IP and IB with antibodies as indicated.

Our results indicated that the M45 protein inhibits TLR and IL-1R signaling to NF-κB by a mechanism that is distinct from its known interaction with RIP1, as IL-1R and TLR2, 7, and 9 signal independently of RIP1 (Figure S1). A plausible explanation for a RIP1-independent mechanism was that the M45 protein interacts with yet an additional target protein within the pathway. This putative interaction partner would be expected to be located downstream of TAK1, because IL-1R-dependent p38 activation was not inhibited by M45 (Figure 1D). The IKK complex lies downstream of TAK1 and is responsible for IκBα phosphorylation that triggers IκBα ubiquitylation and degradation. To determine whether the M45 protein interacts with one of the three IKK subunits, IKKα, IKKβ, or NEMO, we performed co-immunoprecipitation experiments with HA-tagged M45. In these experiments HA-tagged M45 was co-expressed with Flag-tagged versions of IKKα, IKKβ, or NEMO. Immunoprecipitation of M45 with an anti-HA antibody coprecipitated large quantities of NEMO but only small amounts of IKKα and IKKβ (Figure 3A), suggesting that M45 binds to NEMO and interacts only indirectly with IKKα and β via endogenous NEMO. To investigate this possibility, we tried to coprecipitate M45 and IKKα or IKKβ from lysates of NEMO-deficient murine embryonic fibroblasts (MEFs). As shown in Figure 3B, IKKα and β did not coprecipitate with M45 in lysates of *nemo* ^−/−^ MEFs, but did coprecipitate when NEMO was coexpressed. From these experiments we concluded that M45 interacts with IKKα and β only indirectly via NEMO.

It has been shown that polyubiquitylated RIP1 interacts with NEMO [9]. Hence it is conceivable that the M45–NEMO interaction is a result of the known M45–RIP1 interaction. However, the observation that NEMO coprecipitated with M45 in lysates of *rip1* ^−/−^ MEFs (Figure 3C) clearly indicated that RIP1 is not required for the M45–NEMO interaction.

To determine whether M45 also interacts with the endogenous IKK complex, we transfected 293A cells with an expression plasmid encoding HA-tagged M45 or control plasmids. As shown in Figure 3D, endogenous NEMO, IKKα, and IKKβ coprecipitated with M45, but not with an HA-tagged control protein (m143). Similarly, all three IKK subunits could be coprecipitated with M45 from lysates of cells infected with RM45, an MCMV expressing HA-tagged M45 (Figure 3E).

**Figure 4:**
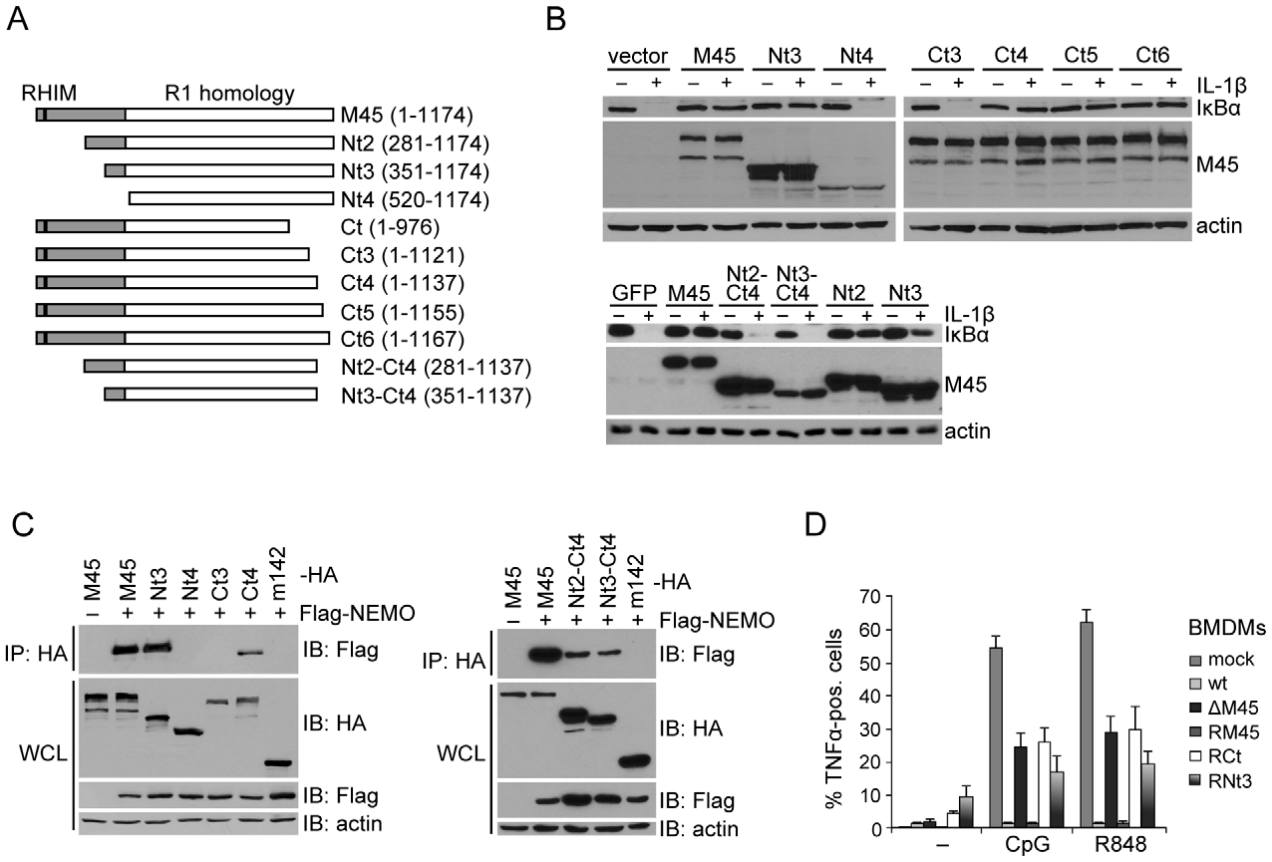
The C-terminal RNR R1 homology domain of M45 is required for the inhibition of NF-κB activation and interaction with NEMO. (A) Schematic representation of M45 truncation mutants used in this and a previous [36] study. The C-terminal RNR R1 homology domain is shown in white, the unique N terminus in grey. The RHIM is marked black. (B) NIH-3T3 cells were transduced with retroviruses expressing full length M45, truncated M45 proteins, or GFP. After stimulation with IL-1β (20 ng/ml, 15 min), IκBα levels were determined by immunoblotting. (C) 293A cells were transfected with plasmids encoding Flag-tagged NEMO and HA-tagged full-length M45, truncated M45, or an unrelated MCMV control protein (m142), respectively. Lysates were subjected to immunoprecipitation (IP) with an anti-HA antibody. Immunoprecipitates and the whole cell lysates (WCL) were analyzed by immunoblotting (IB) with the indicated antibodies. (D) Primary BMDMs were mock infected or infected with GFP-expressing wt MCMV (wt), ΔM45 mutant (ΔM45), M45 revertant (RM45), or MCMVs expressing Ct or Nt3 (RCt and RNt3) at an MOI of 1. 17 h postinfection cells were stimulated for 4 hours with TLR9 agonist CpG (0.5 µM) or TLR7 agonist R838 (0.1 µM) in the presence of brefeldin A. Cells were fixed, permeabilized, and stained with a TNFα-specific antibody. The percentages of TNFα-positive cells within infected (GFP-positive) cell populations were determined by FACS analysis (mean ± SEM).

The M45 protein consists of a C-terminal R1 homology domain and a unique N-terminal domain [39], [40] (Figure 4A). The R1 homology domain has a strong sequence homology to ribonucleotide reductase (RNR) R1 subunits but is devoid of RNR activity [41]. The N-terminal domain, by contrast, contains a RHIM that is required for blocking the RIP1–RIP3 interaction and RIP3-dependent necrosis [37], [38]. To test which part of M45 is required for the inhibition of NF-κB activation, we used a set of N- and C-terminally truncated M45 proteins. These proteins were expressed in fibroblasts using a retroviral vector. Remarkably, up to 350 amino acids of the N-terminus could be deleted without M45 losing its ability to block IL-1β-induced IκBα degradation. By contrast, only 37 amino acids of the C-terminus were dispensable for this activity (Figure 4B). When M45 was truncated at both ends (mutants Nt2-Ct4 and Nt3-Ct4), IκBα degradation was not inhibited (Figure 4B), indicating that the region between amino acids 351 and 1137 is necessary but not sufficient for blocking IκBα degradation. However, this region is both necessary and sufficient for interaction with NEMO in co-immunoprecipitation experiments (Figure 4C).

Two MCMV mutants expressing the truncated M45 proteins Nt3 and Ct, respectively, were used to infect BMDM. Infected cells were stimulated with a TLR7 or a TLR9 agonist, and TNFα production was measured by FACS analysis. As shown in Figure 4D, the full-length M45 protein inhibited TNFα production, but the Ct mutant did not. The Nt3 mutant showed only a partial inhibitory effect, similar to the partial inhibitory effect seen in the IκBα degradation assay (Figure 4B).

### M45 induces NEMO degradation by a ubiquitin–proteosome independent pathway

**Figure 5:**
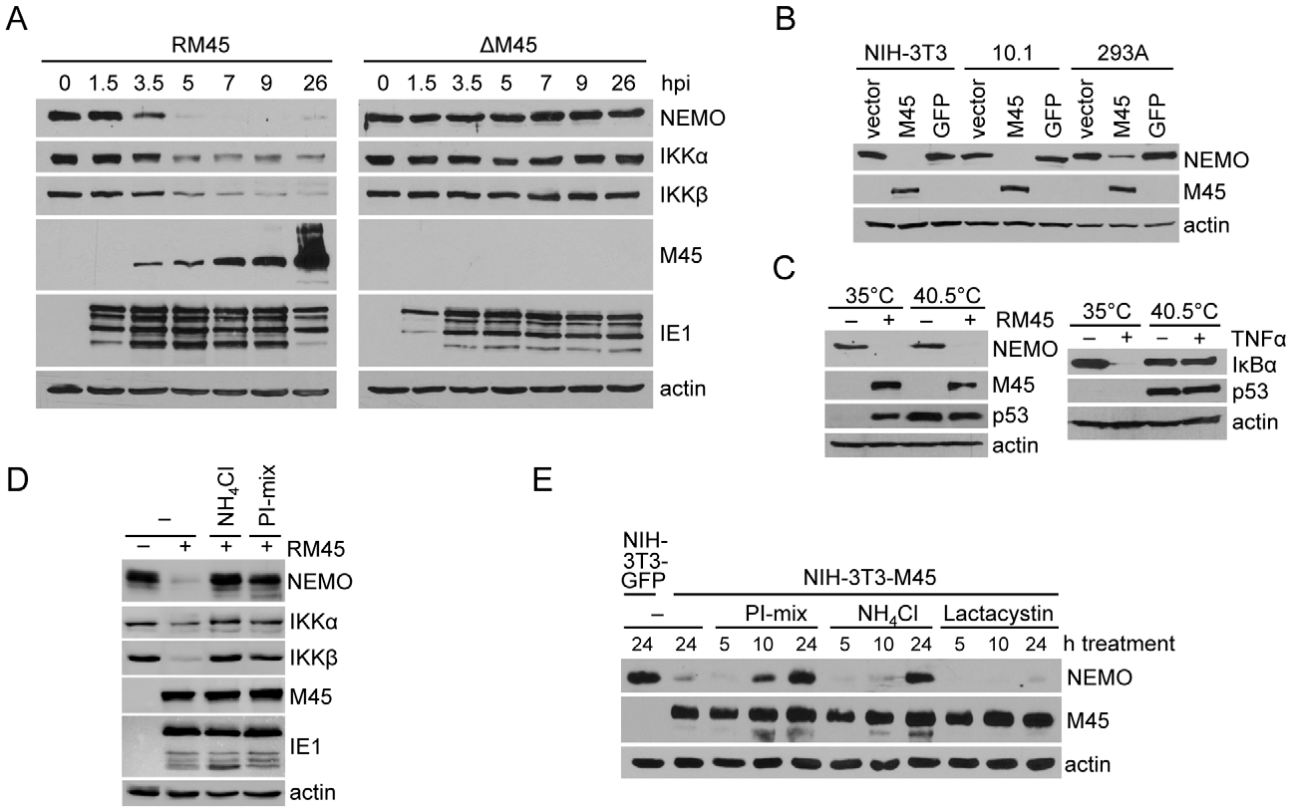
M45 induces lysosomal degradation of NEMO. (A) NIH-3T3 cells were infected with MCMV ΔM45 or the revertant virus, RM45, at an MOI of 3. Cells were harvested at indicated time points postinfection, and the levels of IKK subunits, M45, IE1, and actin in lysates were analyzed by immunoblotting. (B) Murine fibroblasts (NIH-3T3, 10.1) or human embryonic kidney 293A cells were transduced with retroviral vectors expressing M45 or GFP, harvested 72 hours later, and analyzed by immunoblotting. (C) ts20 cells harboring a temperature-sensitive E1 enzyme were incubated for 14 h at a permissive (35°C) or a restrictive (40.5°C) temperature. Cells were then mock-infected or infected with RM45, and analyzed 10 h postinfection by immunoblotting (D) NIH-3T3 cells were mock infected or infected with RM45 (MOI of 5) and treated 2 hpi with 10 mM ammonium chloride (NH_4_Cl) or a mix of lysosomal protease inhibitors (PI-mix). 9 hpi, levels of IKK subunits, M45, IE1, and actin were determined by immunoblotting. (E) NIH-3T3 cells were stably transduced with retroviral vectors expressing M45 or GFP, and treated for the indicated times with PI-mix, 10 mM NH_4_Cl, or 5 µM lactacystin, respectively. Levels of the indicated proteins were analyzed by immunoblotting.

To further explore how the M45–NEMO interaction prevents NF-κB activation, we next analyzed the abundance of the IKK subunits in MCMV-infected cells over time. Strikingly, NEMO started to disappear rapidly as soon as M45 expression began (Figure 5A). The abundance of IKKα and IKKβ also declined in MCMV-infected cells, but these two subunits did not disappear completely. In cells infected with the ΔM45 mutant, the abundance of all three IKK subunits remained largely unchanged over the course of infection (Figure 5A). When M45 was expressed in NIH-3T3, 10.1, or 293A cells using a retroviral vector, NEMO levels were dramatically reduced (Figure 5B), indicating that M45 is both required and sufficient for mediating this effect. Based on these observations we hypothesized that M45 might induce NEMO degradation. First we tested whether the NEMO degradation was ubiquitin-dependent. For these experiments, we used ts20 cells, that have a temperature-sensitive ubiquitin activating (E1) enzyme [42]. The E1 enzyme is active at low temperatures (≤35°C) and becomes inactive at temperatures above 39°C. As shown in Figure 5C, NEMO disappeared in MCMV-infected ts20 cells even at high temperatures. Other proteins, whose degradation is ubiquitin-dependent, such as p53 or IκBα, were stabilized at 40.5°C, indicating that the temperature shift had the expected effect on the ubiquitin system (Figure 5C). Notably, CMV infection itself also prevents p53 degradation by an as-yet undefined mechanism [43].

When NIH-3T3 cells were infected with MCMV, degradation of NEMO and the IKKα and β subunits was inhibited in the presence of a lysosomal protease inhibitor cocktail directly blocking lysosomal degradation or by ammonium chloride treatment that indirectly inhibits lysosomal protein degradation by blocking lysosome acidification (Figure 5D). In NIH-3T3 cells that stably express M45, NEMO levels can also be restored by treatment with lysosomal protease inhibitors or ammonium chloride, but not in the presence of the proteasome inhibitor lactacystine (Figure 5E). Taken together these results indicated that M45 induces NEMO degradation in lysosomes, independent of the ubiquitin-proteasome degradation pathway.

### M45 targets NEMO to autophagosomes

**Figure 6:**
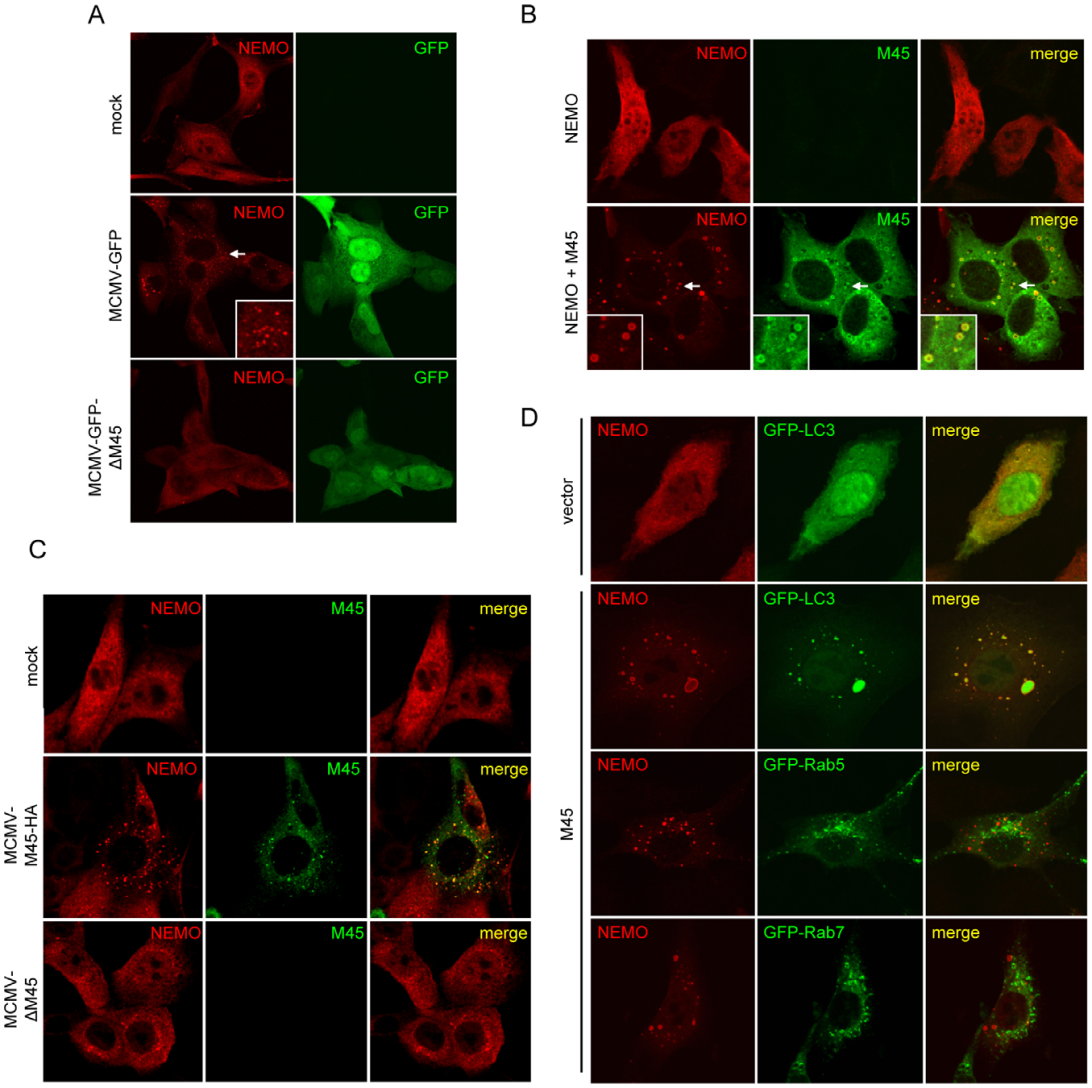
M45 targets NEMO to autophagosomes. (A) NIH-3T3 cells stably expressing Flag-tagged NEMO were mock infected or infected with wt MCMV-GFP or MCMV-GFP-ΔM45 at an MOI of 6. Cells were fixed 6 hpi, and NEMO distribution was analyzed by immunofluorescence. The arrow indicates the region which is shown in higher magnification in the right corner of the picture. (B) NIH-3T3 cells were transfected with Flag-NEMO and M45-HA expressing plasmids as indicated. 24 h later, cells were fixed and double immunofluorescence staining was performed using antibodies against the HA and Flag tags. Regions shown in higher magnification in the left corner of the pictures are indicated by arrows. (C) NIH-3T3 cells stably expressing Flag-NEMO were mock infected or infected with MCMV-M45-HA or MCMV-ΔM45 at an MOI of 7. Seven hpi, cells were fixed and analyzed as described for panel B. (D) NIH-3T3 cells were transfected with expression plasmids for Flag-NEMO and M45-HA or empty vector as indicated. Plasmids encoding GFP-tagged LC3, Rab5, or Rab7 were cotransfected. 24 hours posttransfection cells were fixed and used for anti-Flag immunofluorescence staining.

Degradation pathways often result in the redistribution of target proteins. To gain further insight into the mechanism of NEMO degradation we analyzed the intracellular distribution of NEMO during MCMV infection. NIH-3T3 cells stably expressing Flag-tagged NEMO were infected with a GFP-expressing wt MCMV or an M45 deletion mutant (ΔM45). NEMO was subsequently detected by immuno­fluorescence staining using an anti-Flag antibody. Infection with wt MCMV induced NEMO accumulation in punctate structures as early as 7 hpi. These punctate structures were seen only occasionally in uninfected cells or cells infected with ΔM45 (Figure 6A). NEMO accumulation in punctate or vesicular structures was observed in cells transiently transfected with M45 and NEMO expression plasmids (Figure 6B). M45 also localized to these structures, but was also found diffusely distributed throughout the cytoplasm in transfected (Figure 6B) and MCMV-infected cells (Figure 6C).

Autophagy is a catabolic process involving the lysosomal degradation and recycling of the cell's own components [44]. It is currently the only known mechanism for cytoplasmic proteins to end up in lysosomes. Hence we asked whether the punctate structures, to which NEMO is targeted by M45, represent autophagosomes. GFP-tagged microtubule-associated protein light chain 3 (LC3) is commonly used as a marker for autophagosomes. It is found diffusely distributed throughout the cytoplasm and nucleus in cells with little or no autophagic activity. Upon induction of autophagy, LC3 is conjugated to phosphatidylethanolamine and incorporated into autophagosomal membranes [45], [46]. Indeed, NEMO colocalized with GFP-LC3 in cytoplasmic dots or vesicles when M45 was coexpressed, suggesting that M45 induces NEMO targeting to autophagosomes. By contrast, NEMO did not colocalize with GFP-tagged Rab5 or Rab7, which represent marker proteins for early and late endosomes, respectively (Figure 6D). In MCMV-infected cells, NEMO was also redistibuted to punctate structures that colocalized with GFP-LC3 puncta (Figure S3). NEMO redistribution occurred with and without ammonium chloride treatment.

**Figure 7:**
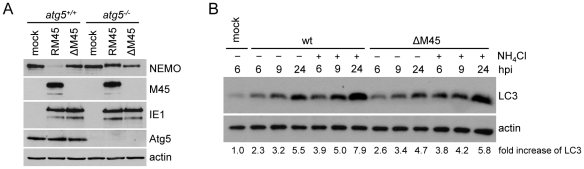
Loss of NEMO degradation in autophagy-deficient MEFs, and MCMV-induced accumulation of LC3-II. (A) *atg5* ^−*/*−^ and *atg5^+/+^* MEFs were mock infected or infected with MCMVΔM45 or the revertant virus, RM45, at an MOI of 10. Eight hpi cells were harvested and levels of the indicated proteins were analyzed by immunoblotting. (B) 10.1 fibroblasts were mock infected or infected with wt MCMV or ΔM45 at an MOI of 5. Cells were left untreated or treated 30 min after infection with 10 mM NH_4_Cl to block lysosomal degradation of LC3-II. Cells were harvested at the indicated time points, and LC3-II levels in cell lysates were analyzed by immunoblotting using an LC3-II-specific antibody. LC3-II levels were quantified by densitometric analysis and normalized using the corresponding actin levels. Fold increases are shown relative to mock-infected cells.

To test whether NEMO degradation is dependent on autophagosome formation, autophagy-deficient *atg5* ^−/−^ MEFs were infected with the MCMV M45 deletion mutant (ΔM45) or the revertant virus, RM45. As shown in Figure 7A, NEMO degradation was inhibited in the absence of ATG5, which is required for autophagosome formation [47].

Next we tested the influence of MCMV infection on autophagosome formation. First we checked whether MCMV infection induced autophagy as it has recently been shown for HCMV and HSV-1 infection [48]. To do this, NIH-3T3 fibroblasts were infected with wt MCMV or ΔM45, and the levels of phosphatidylethanolamine-conjugated LC3 (LC3-II) were determined by immunoblot as an indicator of autophagic activity. Infection with both viruses led to an increase in LC3-II, but this increase was less pronounced in ΔM45-infected cells (Figure 7B), suggesting that M45 enhances but is not solely responsible for autophagosome formation. LC3-II levels were further increased in the presence of a lysosomal inhibitor (Figure 7B), indicating that the infection-induced increase in LC3-II was not due to a block to autophagic flux.

**Figure 8:**
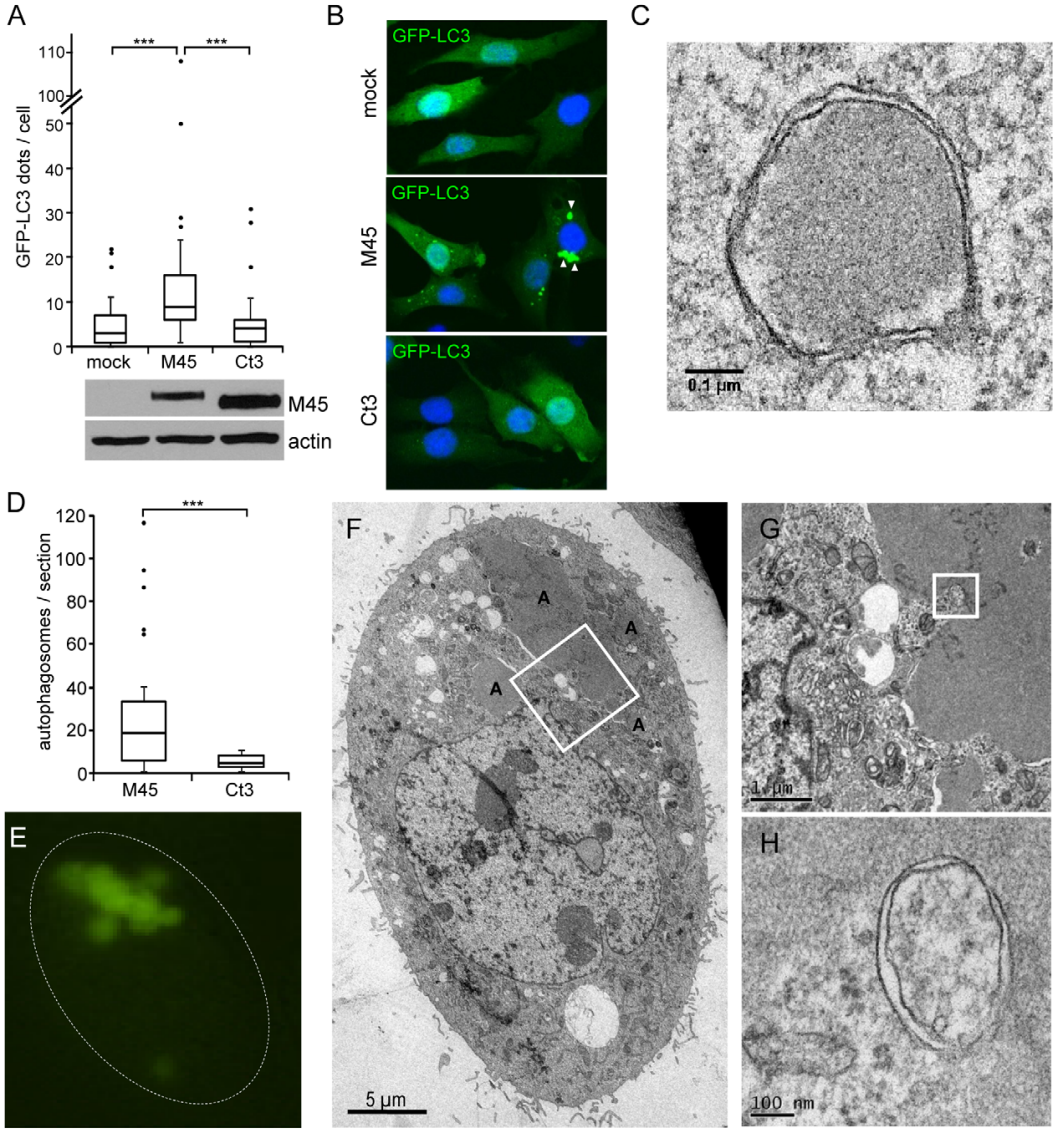
M45 induces the formation of autophagosomes and aggregates. (A) NIH-3T3 cells stably expressing GFP-LC3 were transduced with retroviral vectors expressing M45 or Ct3. Three days later, cells were analyzed by confocal laser scanning microscopy. GFP-LC3 dots were counted in 50 cells per sample. Results are shown as box and whisker plots. The bottom and top of the box represent the first and third quartile, respectively. The horizontal line within the box represents the median. The minima and maxima within 1.5-fold interquartile range are shown as whiskers. Dots represent outliers. Significance was determined using ANOVA (***, p<0.001). M45 and Ct3 were detected by immunoblot. (B) Fluorescence images of cells described above. Large GFP-LC3 aggregates are indicated by arrow heads. (C) Electron micrograph of a double-membrane autophagosome in an M45-expressing cell. (D) Autophagosomes were counted in 10 randomly selected M45- and Ct3-expressing cells (2–3 TEM sections per cell). Results are shown in box and whisker plots as described for panel A. Significance was determined using the student's *t*-test (***, p<0.001). (E) Epifluorescence image of an M45-expressing cell with large GFP-LC3 structures. (F) TEM image of the same cell reveals large aggregates marked with ‘A’. (G, H) Successive enlargement of the region marked in panel F shows an example of an aggregate-associated autophagosome.

The impact of M45 itself on autophagosome formation was subsequently analyzed in NIH-3T3 cells stably expressing GFP-LC3. These cells were transduced with retroviral vectors encoding full-length M45 or the M45 truncation mutant Ct3, which does not bind NEMO (Figure 4). Three days after transduction, cells were fixed and analyzed by confocal microscopy. The number of GFP-LC3 dots per cell section was determined for 50 cells in each population. As shown in Figure 8A, M45-expressing cells contained significantly more GFP-LC3 dots than Ct3-expressing or mock-transduced cells. In addition, many of the M45-expressing cells contained large GFP-positive structures (Figure 8B). Such structures were seen only rarely in the Ct3-expressing or mock-transduced control cells.

GFP-LC3 is a frequently used tool to visualize autophagosomes and monitor autophagy. However, it has been reported that GFP-LC3 can also be incorporated into intracellular protein aggregates, particularly when GFP-LC3 is transiently overexpressed by transfection [49]. Hence we decided to analyze the M45- and Ct3-expressing cells by transmission electron microscopy (TEM). Double-membrane autophagosomes (Figure 8C) were seen regularly in electron micrographs of M45-expressing cells. Similar to the results obtained by fluorescence microscopy (Figure 8A), a detailed analysis of 10 randomly selected M45- and Ct3-expressing cells revealed a significantly higher number of autophagosomes in M45-expressing cells (Figure 8D). In addition, some of the M45-expressing cells also contained large amorphous structures (labeled “A”) that correlated with the large GFP-positive structures in epifluorescence images of the same cells (Figures 8E and F). These large structures probably represent protein aggregates. However, it is noteworthy that autophagosomes were frequently observed adjacent to the putative aggregates (an example is shown in Figure 8F–H), suggesting that autophagosomes are involved in the degradation of these aggregates. By contrast, protein aggregates were detected only rarely in cells expressing the Ct3 protein. Taken together, the results indicate that M45 induces the formation of both, autophagosomes and protein aggregates.

## Discussion

Recognition of microbial pathogens by TLRs and other PRRs results in an activation of NF-κB. This transcription factor is crucial for activating inflammatory and immune responses, as it drives the expression of various cytokines [3]. The inflammatory signaling cascade is amplified by autocrine activation of cytokine receptors, creating a feed-forward loop mediated through the NF-κB pathway (Figure S1). It is well known that viruses have evolved strategies to block signaling pathways leading to NF-κB activation [50]. Phosphorylation of IκB proteins by the IKK complex and their subsequent degradation is the last step before NF-κB translocation to the nucleus and represents the junction point of all classical NF-κB activation pathways. Hence, the IKK complex should be an attractive target for viral intervention.

In this study we showed that the MCMV M45 protein blocks TLR and pro-inflammatory cytokine signaling to NF-κB at the level of the IKK complex, by interacting with NEMO, redirecting it to autophagasomes and inducing its degradation. This mechanism of action has not been previously disclosed and affords the virus the ability to broadly inhibit various receptor-dependent pathways leading to NF-κB activation using a single viral protein. As the NEMO subunit plays a crucial role in all classical NF-κB activation pathways [2], it can be assumed that M45 blocks not only TLR- and IL-1R-dependent NF-κB activation (as shown here), but also NF-κB activating signals emanating from cytosolic PRRs such as the RIG-like helicases and the DNA sensor ZBP1/DAI. As a matter of fact, it has been shown that TLR2, TLR3, TLR7, TLR9, and ZBP1 are activated in the course of CMV infection [21]–[25]. Therefore, a strong selective pressure exists for this virus to acquire such a comprehensive block to the classical NF-κB activation pathway. Moreover, M45 might also attenuate the alternative NF-κB activation pathway, which depends on IKKα but not NEMO [2], as M45 expression during MCMV infection also leads to a substantial reduction of IKKα levels (Figure 5A). This exciting possibility will be investigated in future studies.

A previous study has shown that MCMVs expressing only the first 188 amino acids or less of M45 are highly attenuated in vivo, even in immunodeficient SCID mice [41]. The mutants tested in that study are bona fide M45 *null* mutants lacking M45-mediated inhibition of RIP1 and NEMO. In fact, the previously described region required for interaction with endogenous RIP1 [36] and the region required for interaction with NEMO [this study] seem to be largely overlapping. Once a specific motif required for NEMO but not RIP1 and RIP3 interaction has been identified, M45 could be specifically mutated to abolish NEMO interaction while leaving RIP interactions intact. An MCMV mutant expressing such an M45 protein could then be used to analyze the consequences of uninhibited inflammatory signaling in vivo without losing the ability to inhibit RIP1/RIP3 dependent processes such as necrosis.

In this study we show that M45 induces NEMO degradation by relocalizing the protein to autophagosomes for subsequent degradation in lysosomes. This is a new viral mechanism that has not been described before. Remarkably, the bacterial pathogen *Shigella flexneri* also triggers NEMO depletion, but it does this by inducing its ubiquitylation and proteasomal degradation [51]. Hence, *Shigella* and cytomegalovirus target the same regulatory host protein, but send it to different degradation pathways.

Autophagy is a bulk degradation and recycling system that delivers portions of the cytoplasm and organelles to lysosomes. It contributes to the turnover of large protein complexes and whole organelles, and is strongly increased under starvation conditions in order to maintain an adequate supply of amino acids for protein synthesis [44]. However, more recent evidence indicates that autophagy is also involved in selective degradation of proteins and complexes. For instance, protein aggregates attract factors such as p62/SQSTM1, NBR1, or ALFY, which in turn recruit LC3 and promote autophagosome formation [52]–[54]. In chaperone-mediated autophagy, proteins with a specific amino acid motif are bound by an Hsc70-containing chaperone complex and translocated directly into lysosomes without involvement of autophagosomes [55]. As we show here, M45 targets NEMO to autophagosomes, which are characteristic of macroautophagy. This raises the question of how M45 stimulates autophagic degradation of NEMO. A recent publication showed that the IKK complex contributes to the induction of macroautophagy by a mechanism that is not yet fully understood, but is not dependent on NF-κB activation [56]. Thus, it is conceivable that M45, by interacting with NEMO, coerces the IKK complex to induce its own autophagic degradation. Another possibility is that M45, by interacting with NEMO, displaces the IKK complex from its chaperone, Hsp90 [57]. This could cause NEMO or the entire IKK complex to form insoluble aggregates, which are subsequently degraded by macroautophagy. The formation of large aggregates in cells after prolonged M45 expression and their association with autophagosomes (Figure 8) argues for the latter scenario. Moreover, it has been shown that geldanamycin, a bacterial toxin of *Streptomyces hygroscopicus* that specifically inhibits Hsp90 chaperone function, renders Hsp90 client proteins unstable [58]. Specifically, geldanamycin promotes autophagy-mediated degradation of the IKK complex [59]. Hence, bacteria and viruses seem to utilize overlapping strategies to inhibit crucial signaling pathways of innate immunity.

**Figure 9:**
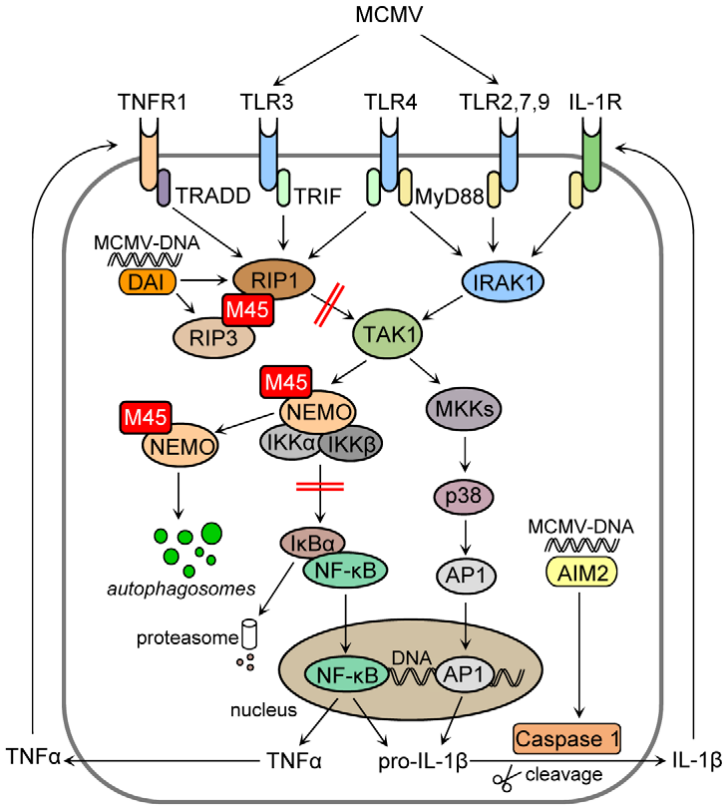
M45 targets RIP1 and NEMO, two crucial components of the inflammatory cascade.

This study combined with previous work [36] shows that the cytomegalovirus M45 protein curtails the inflammatory cascade by targeting two central regulatory proteins, NEMO and RIP1 (Figure 9). Remarkably, NEMO and RIP1 also control signaling cascades in response to the DNA damage sensors ataxia teleangiectasia mutated (ATM) and p53-inducible death-domain-containing protein (PIDD) [60], [61]. As CMV is known to activate ATM-dependent DNA damage responses [62], [63] it can be inferred that M45 may be involved in inhibiting these signaling cascades as well.

## Materials and Methods

### Antibodies

We used the following antibodies: Monoclonal antibodies against IKKβ (2C8, Cell Signaling), phopho-p38 (3D7, Cell Signaling), Flag (M2, Sigma), β actin (AC-74, Sigma), RIP1 (38, BD Transduction Laboratories), HA (16B12, Covance Inc.), HA (3F10, Roche), NF-κB p65 (F-6, Santa Cruz, or C22B4, Cell Signaling), LC3B (D11, Cell signaling), IE1 antibody (CROMA101; provided by Stipan Jonjic, University of Rijeka, Croatia) and polyclonal antibodies against NEMO (FL-419, Santa Cruz), IKKα (M-204, Santa Cruz), p38 (C-20, Santa Cruz), IκBα (C-21, Santa Cruz), p53 (FL-393, Santa Cruz), Atg5 (Sigma), HA (Sigma), Flag (Sigma), M45 ([41] provided by David Lembo, University of Turin, Italy). Secondary antibodies coupled to HRP were purchased from Dako.

### Inhibitors, ligands, and reagents

Lysosomal protease inhibitors were purchased from AppliChem and used in the following concentrations: E-64, 20 µM; bestatin hydrochloride, 72 µM; leupeptin, 100 µM; pepstatin A, 3.64 µM. Lactacystin was purchased from Calbiochem, ammonium chloride from Merck, and Brefeldin A from Sigma. The following receptor ligands were used: LPS, LTA-SA, CpG (ODN 1668 or ODN 1826), R837 (Invivogen), R848 (Enzo LifeSciences), IL-1β, Pam_3_CSK_4_ (Imgenex), Malp-2 (Alexis Biochemicals), TNFα (Promokine).

### Plasmids

The following plasmids were purchased from Addgene: pCMVTAG-NEMO, pCR-Flag-IKKα, pCMV2-Flag-IKKβ, pEGFP-LC3, and pEGFP-Rab7. Plasmids pNiFty2-SEAP (Invivogen), pcDNA3 (Invitrogen), pEGFP-C1 (Clontech), pcDNA-EGFP-Rab5 (provided by Kira Gromova, Freie Universität Berlin, Germany), pcDNA-m142-HA, -m143-HA, and -m143-Flag [64] were obtained from sources as indicated. pcDNA-M45-HA and the truncation mutant Nt3 has been described [36]. Truncation mutant Nt4 was created by digesting pcDNA-M45-HA with *Box*I and *Kpn*I, blunting and religation. Truncation mutants Ct3 through Ct6 were generated by PCR, replacing the *Bsu*36I–*Apa*I fragment of pcDNA-M45-HA. Truncation mutant Nt2-Ct4 was generated by digesting pcDNA-Ct4 with *Hind*III and religation. Truncation mutant Nt3-Ct4 was generated by digesting pcDNA-Ct4 with *Kpn*I and *Eco*RI, blunting, and religation**.**

### Cells and viruses

NIH-3T3 (ATCC CRL-1658) and 10.1 [65] cells are immortalized MEFs. Immortalized *rip1* ^−/−^ MEFs were kindly provided Michelle Kelliher (University of Massachusetts, Boston, MA), *nemo* ^−/−^ MEFs by Michael Karin (University of California, San Diego), and ts20 cells by Robert Kalejta (University of Wisconsin, Madison). Human embryonic kidney (HEK) 293A cells were purchased from Invitrogen. Murine RAW264.7 macrophages were obtained from ATCC (TIB-71). Immortalized *atg5* ^−*/*−^ and *atg5^+/+^* MEFs were purchased from RIKEN BRC cell bank. Bone marrow-derived macrophages (BMDMs) were derived from bone marrow of C57BL/6 mice as described [66].

MCMV-GFP, the M45 deletion mutant (ΔM45), the M45 revertant virus (RM45), and an MCMV expressing HA-tagged m142 (Rm142) have been described [36], [39], [67], [68]. RNt3 and RCt were constructed in the same way as described for RM45 [36], except that truncated M45 sequences instead of full-length M45 were inserted into ΔM45. All these MCMV mutants express GFP. In addition, an M45 deletion mutant was also constructed on the basis of the GFP-less wt MCMV [69] in the same way as described for the GFP-expressing ΔM45 mutant [67]. MCMV-M45-HA was constructed by inserting an HA tag sequence at the 3′ end of the M45 ORF essentially as described [67]. MCMV-M45-HA does not express GFP. Viruses were grown and titrated on NIH-3T3 cells according to standard procedures. Viral titers were determined using the median tissue culture infective dose (TCID_50_) method.

### Retroviral transduction

pRetroM45, pRetroGFP, and the empty retroviral vector plasmid have been described [36]. M45 truncation mutants Ct3, Ct4, Ct5, and Ct6 were excised as *Kpn*I/*Apa*I fragments from the pcDNA3 vector, blunted, and insertion into the *Pml*I site of the retroviral vector plasmid. The Nt2 and Nt3 sequences were excised with *Hind*III/*Apa*I *and Xba*I/*Eco*RI, respectively, blunted, and inserted in the same way. The Nt4 sequence was PCR-amplified and inserted between the *Bam*HI and *Eco*RI sites of the retroviral vector plasmid. The Nt2-Ct4 and Nt3-Ct4 sequences were excised from pcDNA-Ct4 with *Apa*I and *Hind*III or *Eco*RI, respectively, blunted and inserted into the *Pml*I site of the retroviral vector plasmid. Flag-tagged NEMO was excised with *Not*I and *Xho*I from pCMVTAG-NEMO and inserted into pMSCVpuro (Clontech). pBABEpuro-GFP-LC3 (Plasmid 22405) was purchased from Addgene. Retroviruses were generated using the Phoenix packaging cell line and used to transduce target cells as described [70].

### NF-κB reporter assay

pNiFty2-SEAP (Invivogen) is an NF-κB-inducible reporter plasmid expressing Secreted Embryonic Alkaline Phosphatase (SEAP). NIH-3T3 cells harboring this construct were generated by transfecting cells with *Not*I-linearized pNiFty2-SEAP followed by selection with zeocin. NIH-3T3-NiFty2-SEAP cells were seeded on 96-well plates and transduced 24 h later with retroviral vectors. Three days after transduction, cells were stimulated with IL-1β (5 ng/ml), Pam_3_CSK_4_ (1 µg/ml) or LPS (0.1 µg/ml). SEAP activities in supernatants were determined photometrically 15 h later using QUANTI-Blue detection reagent (Invivogen) according to the manufacturer's protocol. Results are shown as fold induction of SEAP secretion of stimulated cells compared to unstimulated cells with standard deviation (SD).

### Immunoprecipitation and immunoblotting

For immunopreciptiation cells were grown in 10 cm dishes and transfected with Polyfect (Qiagen) or by the calcium phosphate precipitation method. Cells were harvested 24 to 48 h later with lysis buffer [50 mM Tris-HCl pH 7.5, 150 mM NaCl, 1% Nonidet P-40 and complete protease inhibitor cocktail (Roche)]. Insoluble material was removed by centrifugation. Proteins were precipitated using anti-HA or anti-Flag antibodies and protein A or protein G Sepharose (GE Healthcare), respectively, washed 6 times, eluted by boiling in sample buffer, and subjected to SDS-PAGE and immunoblotting.

For protein expression kinetics, cells were lysed in RIPA buffer (20 mM Tris-HCl (pH 7.5), 300 mM NaCl, 1% sodium deoxycholate, 1% Triton X-100, 0.1% SDS, complete protease inhibitor cocktail). For all other immunoblot analyses, cells were lysed in boiling SDS PAGE sample buffer.

### Immunofluorescence

NIH-3T3 cells were grown on coverslips, washed with PBS, and fixed for 20 min in 4% paraformaldehyde in PBS. Cells were incubated with 50 mM ammonium chloride, permeablized with 0.3% TritonX-100, and blocked with 0.2% cold-water fish skin gelatin (Sigma). Cells were then incubated with primary antibodies for 1 h at room temperature (RT) or overnight at 4°C, washed three times with PBS, and incubated for 1 h with secondary antibodies coupled to AlexaFluor594 or AlexaFluor488 (Invitrogen). Samples were washed, mounted on slides with Aqua-Poly/Mount (Polysciences), and analyzed by confocal laser scanning microscopy using a Zeiss LSM510 Meta microscope.

BMDMs were treated similarly with the following exceptions: BMDMs were seeded onto microdot slides (C.A.Hendley Ltd.), blocking was done with PBS/5% normal goat serum, and ProLong Gold antifade reagent with DAPI (Invitrogen) was used for mounting.

### Correlative transmission electron microscopy (TEM)

For correlative TEM, cells were grown on culture dishes with imprinted grids (Ibidi) in order to localize target cells identified by epifluorescence microscopy. Cells were fixed with 2.5% glutaraldehyde in PBS for 30 min at room temperature. Subsequently, cells were washed with PBS, postfixed for 30 minutes with 1% OsO_4_ in PBS, washed with ddH_2_O, and stained with 1% uranyl acetate in water. The samples were gradually dehydrated with ethanol and embedded in Epon resin for sectioning. Single cells were stamped out and sectioned parallel to the plain of the culture dish. Ultrathin sections (50 nm) were prepared using an Ultracut Microtome (Reichert Jung). All sections were counterstained with 2% uranyl acetate and lead citrate. Electron micrographs were obtained using a Philips CM 120 TEM at 80 kV and a Gatan Multiscan 794 camera.

### Cytokine detection by intracellular TNFα FACS staining and ELISA

Infected RAW264.7 macrophages or primary bone marrow derived macrophages were stimulated for 4 hours with TLR agonists (Pam_3_CSK_4_, 1 µg/ml; Malp-2, 0.1 µg/ml; LPS, 1 µg/ml; R837, 10 µM; CpG-ODN1826, 1 µM) in the presence of 10 µg/ml brefeldin A. Cells were trypsinized, fixed with 3.7% formaldehyde in PBS for 10 min at RT, and permeabilized with 0.5% saponin in FACS buffer (PBS with 2% inactivated fetal calf serum) for 20 min at RT, stained with an AlexaFluor 647-conjugated anti-mouse TNFα antibody (MP6-XT22, BD Pharmingen) for 30 min at RT, washed, and analyzed by FACS. IL-6 and TNFα secretion into culture medium by BMDMs was measured using commercial ELISA kits (R&D Systems), following the manufacturer's instructions.

## Supporting Information

Figure S1 Simplified diagram of TNFR1-, TLR-, and IL-1R-dependent feed-forward signaling pathways to NF-κB and p38 activation.
(TIF)

Figure S2 M45 inhibits TLR-dependent NF-κB activation in primary macrophages. BMDMs were mock infected or infected with wt MCMV or ΔM45, and stimulated with TLR7 and TLR9 agonists R848 and CpG, respectively. NF-κB p65 and the viral immediate-early 1 (IE1) protein were detected by immunofluorescence staining. Nuclei were counterstained with DAPI. Note that not all cells are infected, but only those expressing IE1.
(TIF)

Figure S3 NEMO redistribution upon MCMV infection independent of NH_4_Cl treatment. NIH-3T3 cells stably expressing Flag-NEMO were transfected with a GFP-LC3 expression plasmid and infected 48 hours later with wt MCMV at an MOI of 10. Eight hpi cells were fixed and subjected to immunofluorescence staining using an anti-Flag antibody.
(TIF)
