# Supplementary figures and images for: Viral Mediated Redirection of NEMO/IKKγ to Autophagosomes Curtails the Inflammatory Cascade
Source: PLoS Pathog. 2012 Feb 2;8(2):e1002517. doi: 10.1371/journal.ppat.1002517 (PMC3271075; doi:10.1371/journal.ppat.1002517)

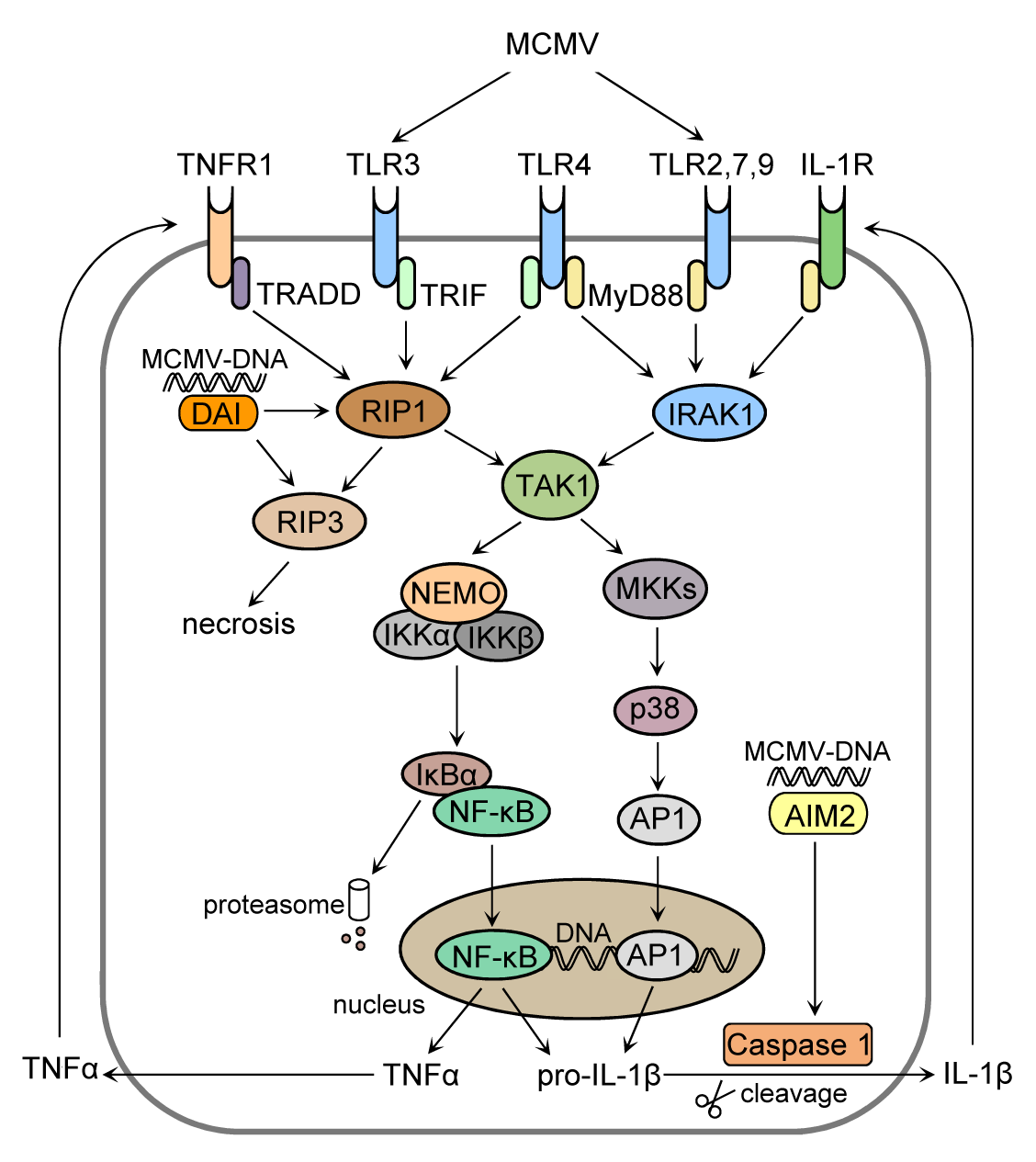

Supplement: Figure S1 — Simplified diagram of TNFR1-, TLR-, and IL-1R-dependent feed-forward signaling pathways to NF-κB and p38 activation. (TIF) [file ppat.1002517.s001.tif]

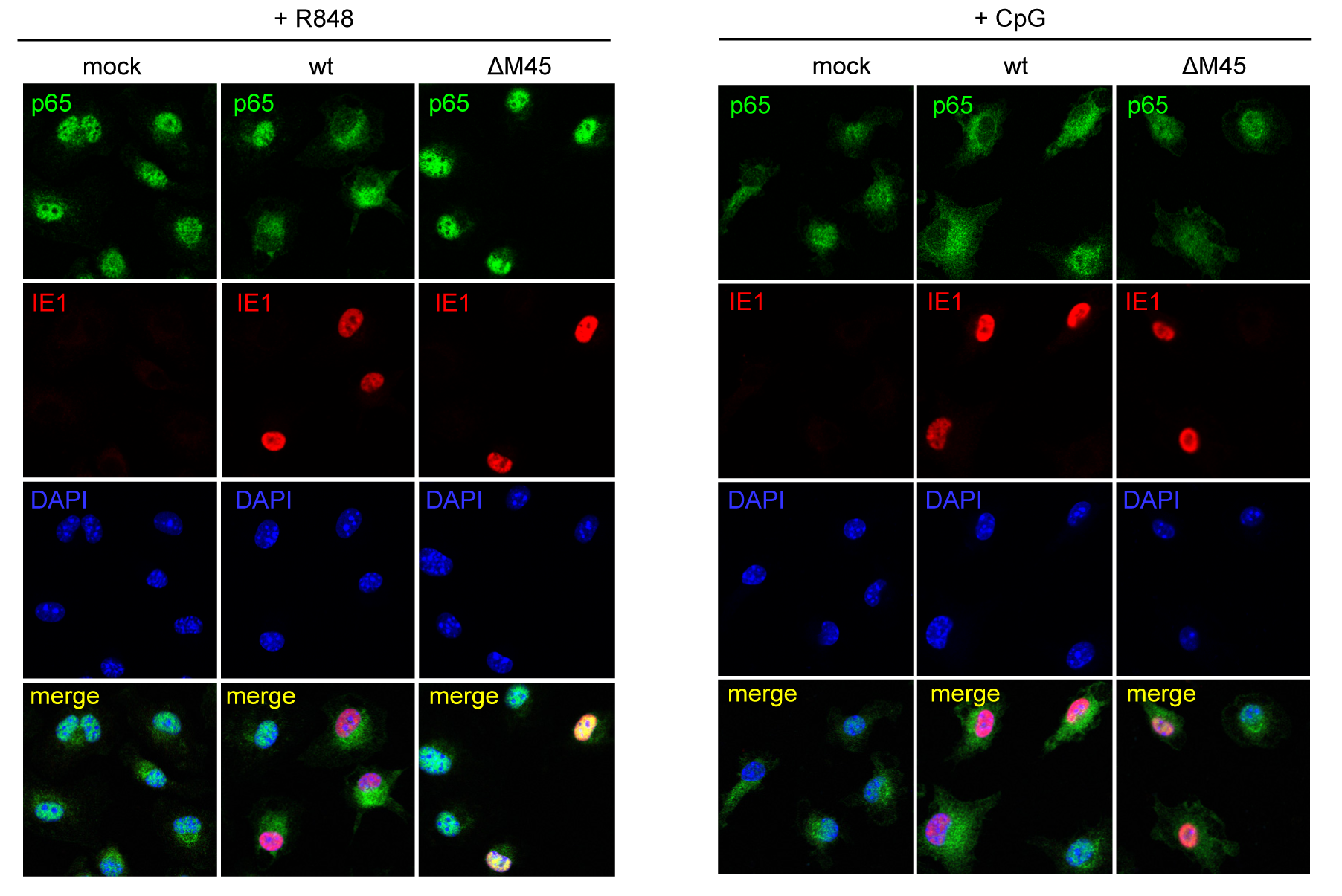

Supplement: Figure S2 — M45 inhibits TLR-dependent NF-κB activation in primary macrophages. BMDMs were mock infected or infected with wt MCMV or ΔM45, and stimulated with TLR7 and TLR9 agonists R848 and CpG, respectively. NF-κB p65 and the viral immediate-early 1 (IE1) protein were detected by immunofluorescence staining. Nuclei were counterstained with DAPI. Note that not all cells are infected, but only those expressing IE1. (TIF) [file ppat.1002517.s002.tif]

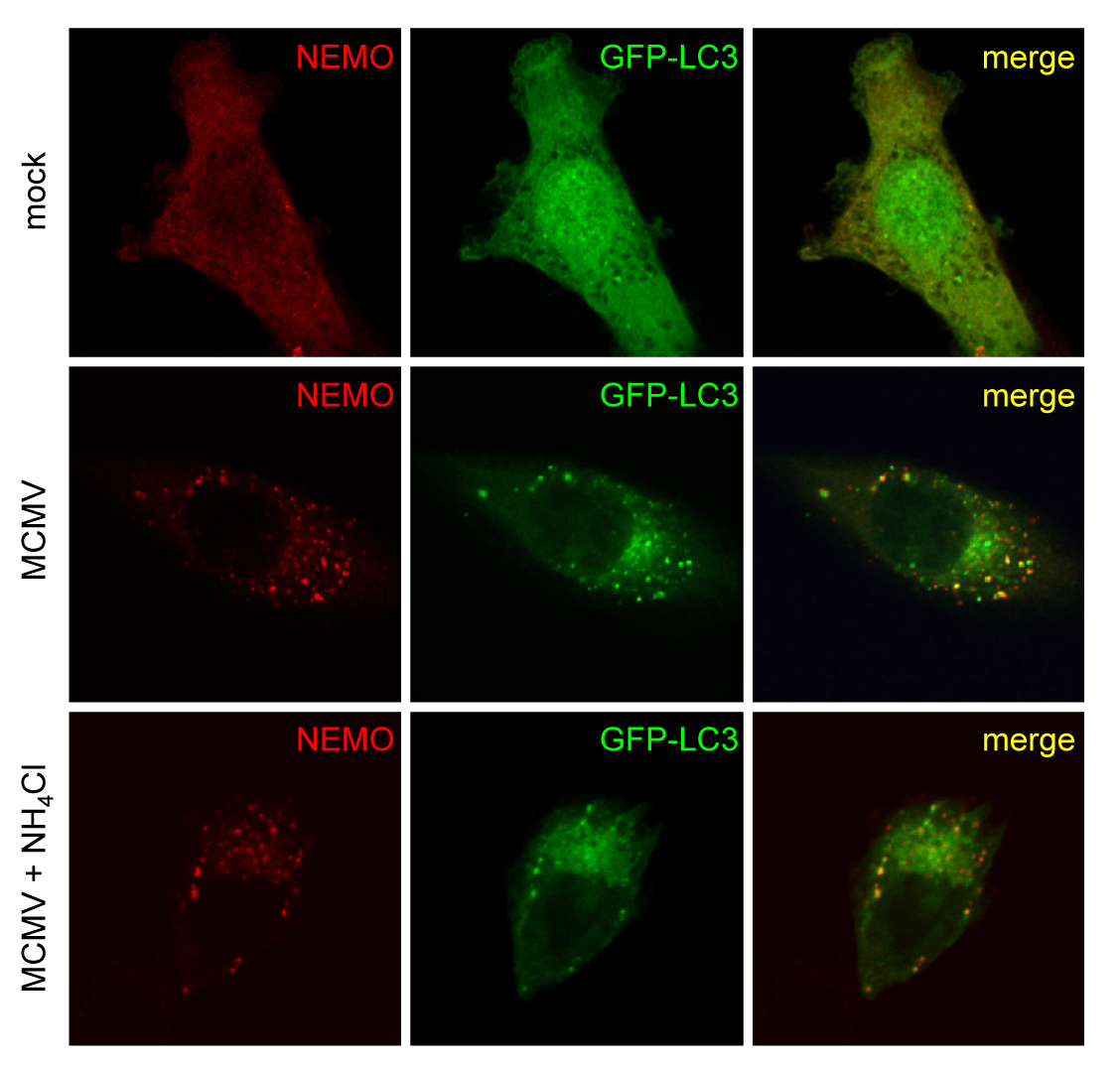

Supplement: Figure S3 — NEMO redistribution upon MCMV infection independent of NH4Cl treatment. NIH-3T3 cells stably expressing Flag-NEMO were transfected with a GFP-LC3 expression plasmid and infected 48 hours later with wt MCMV at an MOI of 10. Eight hpi cells were fixed and subjected to immunofluorescence staining using an anti-Flag antibody. (TIF) [file ppat.1002517.s003.tif]
